# Extracts and Gleanings

**Published:** 1875-12

**Authors:** 


### Extracts and Gleanings.

---

*Sulphate of of Cinchonidia in Intermittent Fever.*—Dr. Parvin (*American Practioner*) says: Nearly two months ago I commenced the use of sulphate of cinchonidia. It was administered in a solution* made by adding to one ounce of the salt two fluid ounces of aromatic sulphuric acid and fourteen fluid ounces of water. A teaspoonful or one fluid drachm of this solution would, therefore, represent three

*Since writing the above, some three weeks ago, many new cases of "intermittent" have occurred at the Reformatory, and they have been characterized by greater gastric disturbance and greater obstinacy to the action of remedies. For the former reason I have replaced the solution mentioned by pills of the sulphate of cinchonidia, tartaric acid, and water (this is an admirable way of making pills); and for the latter I have given the sulphate in larger doses ; in one instance forty grains within twelve hours. The general result has been still quite satisfactory. In the few instances where the remedy failed to arrest the disease it was not given in sufficient quantity, or was rejected by vomiting.

grains and three-quarters of the sulphate. The number of cases of intermittent fever treated with this preparation was twenty-four: two quotidians, one quartan, and the rest tertians. Immediately upon the manifestation of the disease, two teasponfuls of the solution previously mentioned were given, and continued three times—adults four times—a day for three days; then, in order to prevent a relapse, two teaspoonfuls twice a day for one month. In no instance did it fail to promptly arrest the disease, and in no case has there been a recurrence, save when there was a failure to take the remedy as directed. In a case of Wood's *pernicious fever* I did not think it right to risk the cinchonidia, but gave quinia; nor have I yet tried it in malarial neuralgia, having had such long-established confidence in the value of quinia with morphia and the English extract of belladonna in this disorder; but, with these exceptions, the sulphate of cinchonidia is almost the sole reliance as an anti-periodic in my practice at the Reformatory.

*Precaution Against Puerperal Infection.*—At a meeting of a medical society in St. Petersburg (*Rundschau*) Dr. Grünewaldt detailed the measures which were adopted at the lying-in asylum of that city to prevent infection. Acting on the theory that some wound or laceration of the parts concerned in labor presented a nidus for the reception of the disease, attention was also directed toward these points, and the results had been happy. The most common starting-point was held to be the vagina or the mouth of the womb. At these points the poison was absorbed, and the disease traveled onward along the planes of connective tissue. The rule observed was to examine every woman, by the aid of a speculum, immediately after labor. Every laceration or abrasion was then carefully attended to, and as soon as a morbid appearance, such as diphtheritic deposit or the like, was noticed, a solution of carbolic acid in water (one part to twelve) was applied, or the sesquichloride of iron in a similar proportion. If the disease advanced, and there were signs of endometritis, injections were practiced by means of the double catheter. The indications for intra-uterine injections were (1) retention of the membranes, (2) retention and decomposition of coagulated blood, (3) in lochiometra, (4) in any form of endometritis, and (5) in secondary hemorrhage. In fact, he said it was customary to use a weak solution of carbolic acid (one part to four hundred) as an intra-uterine injection immediately after labor, in all cases, by way of prophylaxis.

*Arnica in Orchitis.*—Mr. H. G. Knaggs reports a method of treating orchitis which, he says, he has for many years found very effective. It consists in the more or less constant application, while the patient is resting, of a lotion of tincture arnica and water (one part of the former to six of the latter) to the affected organ; secondly, in rubbing in an embrocation composed of one third or even one half tincture of arnica and soap-liniment, two or three times a day, along the course of the spermatic cord; and thirdly, in the internal administration of seven-drop doses of tincture of arnica, combined when there is febrile disturbance with two-and-a-half-drop doses of Fleming's tinc. of aconite and acetate of ammonia. This simple treatment, he says, generally cures the patient in a fortnight or less. In using our remedial agents of the above-named strength there is little danger of causing cutaneous irritation; but it must be admitted that while some skins will bear the constant application of even pure tincture of arnica for a considerable time, there are others which are inconceivably sensitive to the action of the drug. We must therefore be on the watch for any show of erysipelatoid inflammation in case such occur.—*British Medical Journal.*

*Production of Meteorism.*—Mr. Leven has reported to the Biological Society of Paris the result of various experiments which he has made on animals respecting the composition, production and effects of gases in the stomach and small intestines. His conclusions, which are of much interest in elucidating the pathology of dyspepsia, are opposed to some generally entertained opinions. When an animal is opened after death gases are found in the intestines, and physiologists say these gases proceed from the reaction of the alimentary substances on one another. M. Leven maintains that this explanation is incorrect; that no alimentary substance ferments; and that the term "ferment" applied to pepsin is not exact, as pepsin does not act in the manner of a ferment, but has a fixed action, and modifies the physical state of the alimentary substances so as to render them absorbable. The fluids of the digestive tube preserve but do not decompose the alimentary substances. M. Leven, in his experiments upon dogs, has never found inflammable gas; the only gases he has found, being oxygen, nitrogen and carbonic acid. He has never failed to find nitrogen, but oxygen and carbonic acid have been in some instances entirely absent. The same gases have been found in the stomach and in the small intestines. Applying these results, M. Leven considers that the production of meteorism is not alone due to gas, but to paralytic distension of the muscular fibres of the stomach and intestines, produced by the introduction of hard, ligneous, indigestible matters, which irritate the muscular system even before digestion commences. To demonstrate this he introduced air into the digestive organs of a dog; the animal was seized with dyspnea and muscular trembling, but recovered in half an hour. M. Leven deduces from this fact that alimentary substances do not of themselves change either the nature or quanity of the gases in the stomach or intestines; and that as in flatulent dyspepsia the gases do not result from alimentary decomposition, the principal cause of the phenomena observed is paralysis of the muscular tunic of the digestive tube.—*Gazette Hebdom.*

*Chloral and Bromide of Potassium in Enema in Diseases of Women.*—Dr. G. de G. Griffith advises the administration of chloral and bromide of potassium in enema, as nausea and the burning or disagreeable taste is thus obviated, and the gastric nerves are not affected. As irritating sensation in the rectum may be avoided by beating up the drug with one or two eggs and adding one or two eggs and adding a little warm milk. He records a case of violent puerperal madia successfully treated with nutrient injections, to which he added a drachm of bromide of potassium and half a drachm of chloral. In a case of gall-stones occurring in the person of a lady whose stomach was so irritable that nothing could be retained, and with whom all other remedies failed, injections of chloral in half-drachm doses were successful. In cases of menstrual pain and sickness, in the uterine and ovarian irritation, and in irritable conditions of the rectum, enemata of chloral have been found most efficacious.—*British Medical Journal.*

*Threatened Death During the Administration of Ether.*—Dr. Frederick Haynes, of Leamington, writes to us: Some few months ago a patient to whom I was administering ether nearly died. The ether used was pure sulphuric ether. The patient was a girl, aged fourteen; and the aæsthetic was administered for an operation for necrosis of the tibia (Mr. Marriott operating). A fluid ounce was poured on a sponge, wrung out of hot water, placed in a leather cone, and was given with as little air as possible when first administered. The patient had been under the influence of ether for about ten minutes, when she suddenly became blue in the face, and the jaws closed with a spasm, and the pulse could not be felt at the wrist. I immediately called the attention of the staff to her condition; pulled out her tongue and turned her quickly on her left side, that being the readiest method for artificial respiration. She gave a few gasps, vomited, and eventually recovered. I report this case as it shows ether not to be free from danger, even when pure sulphuric. Though I have often administered chloroform, and ether pretty frequently, I never had a case which gave me so much anxiety. The patient was anæsthetized and ready for operation in two minutes.—*American Medical Weekly.*

*Liniments of Croton Oil.*—

℞.—Olei croton tiglii, . . ʒj.

Ether sulph., . . . ʒij.

Tinct. iodine, . . . ʒv.

M. To be applied two or three coats at a time, with a camel's-hair brush, over a small surface, once a week. This is most useful for children, females, and sensitive males. The stronger croton oil paint is given as follows:

℞.—Olei croton tiglii, . . ʒij.

Ether sulph., . . . ʒiv.

Tinct. iodini, . . . ʒij.

Potass. iod., . . . . ℈j.

Iodine, . . . . x grs.

M. Paint as before.

These croton oil paints are advocated, by Dr. John W. Corson, as a substitute for blistering, in all cases where a counter-irritant effect is desired. They produce less debilitating effect, and are more steady and continuous in their effects.

*Double Fistula in Ano; Treatment by the Knife and Elastic Ligature.*—At a recent meeting of the Clinical Society of London, Mr. Maunder reported the above case, as a test of the relative value of the elastic ligature, recommended by Mr. Allingham, as compared with the knife. The patient was a female, twenty-four years of age, in whom a fistula had formed in the right side of the anus from abscess of the buttock, and a year later a similar one formed on the left side. The two sinuses were almost exactly alike, both as regards position and direction, and therefore well adapted for the comparative trial. The right was laid open with the knife, and dressed with oiled lint, and the left treated with the ligature. On the ligatured side there was much pain, but scarcely any on the right side, the knife wound healing rapidly. The ligature came away on the ninth day, leaving a wound with callous prominent edges, which did not cicatrize until five weeks after the side treated by the knife, although in the latter the lint had been left in place forty-eight hours longer than usual. Mr. Maunder believed that the ligature should only be used in those cases where there is objection to a cutting operation, and in those who are the subjects of a hemorrhagic diathesis.

In the discussion which followed, Mr. Heath said that where the fistula was seated high up, in order to avoid the hemorrhage, it would be more prudent to use the elastic ligature or a similar method, *e.g.*, Mr. Luke's (the use of an ordinary ligature, daily tightened). Mr. Hutchinson could confirm Mr. Maunder's statement as to the slowness of healing after the use of the ligature. Mr. Hulke had never used the ligature himself, but had seen it employed in three cases, but the extreme pain and the slow progress made in each case had convinced him of the inferiority of the method.

Mr. Thomas Smith had twice tried the elastic ligature, the result being that he would never resort to it again, unless prevented by very good reasons from using the knife.—*The Lancet.*

*Inunction in Typhoid Fever.*—Dr. Thompson (*Medical Record*) says: A measure which I adopt in every case of severe fever of any type, but notably in the exanthemata—and that is, general inunction of the body several times a day either with simple oliveoil or with the lin. aq. calcis, composed of equal parts of olive-oil and lime-water. This is one of the oldest means of therapeutics, and the longer I employ it the more I am satisfied that it confers several peculiar and positive benefits; but notably in those complaints in which the skin itself becomes severely inflamed, as in small-pox and scarlatina. But its advantages in other fevers also are not slight. In the first place it greatly allays the irritability of the surface nerves, which is such a source of restlessness and shifting of the position in fevers. It calms the nervous centres themselves when disturbed by a too constant transmission of external impressions by the excited peripheral nerves, and hence is no mean adjuvant towards procuring sleep. By relieving the tension of the skin also, it disposes to a gentle perspiration, and to that fact I attribute the still further advantage which is undoubtedly often gained by it, namely, of lowering the temperature of the body. You will frequently note a fall of from half a degree to a full degree in the thermometer under the tongue half an hour after a general oiling, and which, therefore, cannot be ascribed to a mere cooling of the surface by exposure. Another effect which I have noticed is, that it relieves thirst, which I would ascribe to its restoring a function of the skin, which enables it to add water to the system when needed, as well as to abstract it when the circulation is too full, and the arrest of which function I regard to be the cause of the thirst always present when the skin is rendered dry either by fever or by the tension of general anasarca. In every case of typhoid, therefore, I direct that the patient be rubbed with oil by the palm of the hand three times a day, and oftener if there be great restlessness or the skin be very hot, and the attendants very soon perceive the use of persevering in the applications on noting the patient often coming out of his delirium during its performance. By this means, also, the vitality of the skin is maintained in those parts where you are liable to have bed-sores, so that you will rarely be troubled with this unpleasant complication.

*Dressing for Ulcers.*—℞. Olei cadini, ℥ss ad ℥i; pulv. calci sulphat, ℥vj. M. To be thinly spread on dressings for ulcers, and renewed three, four, five, or six times daily, taking care to cleanse the ulcers after the dressing has been removed. Of special value in the treatment of profuse and fetid suppuration.

*Treatment of Cerebral Rheumatism by Hydrate of Chloral—New Method.* (From report to Academy of Sciences.)—M. Bouchut communicated the following note:

The displacement of acute articular rheumatism and its transfer to the membranes of the brain, called cerebral rheumatism or rheumatismal meningitis, is always attended with great danger.

Pathological anatomy and ophthalmoscopy prove that this complication of acute articular rheumatism is a form of meningitis. Examination of the membranes of the brain reveals a considerable venous stasis, with an opaline infiltration of the pia mater—caused by numerous lucocythes.

The ophthalmoscope, that permits us to follow in the fundus of the eye the developments of the alterations going on in the cerebral substance and in the meninges, shows a serious infiltration of the papilla and of the adjoining portion of the retina, with dilatation of the retinal veins, which represent the similar alterations of the pia mater and of the brain.

The rheumatism of the brain manifests itself by delirium, more or less violent, terminating in coma and in asphyxia, often so rapid as to cause death in several hours.

In three cases of this nature the patients were cured by the hydrate of chloral taken by the mouth, in quantity of three to six grammes, in one or two doses, one rapidly following the other, so as to quickly calm the agitation under which such patients labor.

*Russian Cure for Drunkenness.*—H. Haurowiz says that for sometime past *herba serpylli* (wild thyme) has been used with great success to effect a permanent cure of drunkenness; in case of a relapse, (only after years,) a short treatment will effect a cure again. The treatment consists in making an infusion of wild thyme, (1½ ounces to 1½ pints,) and give the patient a teacupful every half hour. The next day it is given every two hours, and then four to six times a day until the cure is complete, which generally takes from two to three weeks. The effects are in the following order: vomiting, diarrhœa, increased urine, strong transpiration; then, generally, increased appetite, and craving for acidulous beverages. Diet: easily digested food, and lemonade or other acidulous liquids. *American Journal of Pharmacy.*

*Causes of Addison's Disease.*—Dr. E. H. Greenhow (*British Medical Journal,* March), in a series of lectures, gives a considerable number of interesting facts bearing on the study of Addison's disease.

From these facts it appears that this disease takes place almost exclusively in persons employed in active manual labor.

The mortality caused by it is pretty equally distributed over the laborious period of life, and to that period it is almost exclusively confined.

The disease is comparatively much more frequent in persons of the male sex, whose employment naturally involves the heaviest kinds of labor.

A preponderating number of the cases which occur in persons of the male sex are found among those classes of laborers whose occupations are most likely to expose them to bodily injury from accident or over-exertion.

From these and other analogous facts it would seem that in many cases Addison's disease was due to traumatic causes, though its development may have been aided by certain constitutional proclivities.

*Bismuth and Creasote in Infantile Vomiting* (*British Medica Journal,* September 25, 1875).—Dr. Edward Mackey has for a number of years used the following method of treatment in cases of infantile vomiting, and with great success.

Purgatives or ordinary astringents being either premised or contra-indicated, a valuable remedy is known in quarter or half-drop doses of dilute hydrocyanic acid, with a grain or two of soda in camphor or dill water. But in severe cases with much depression, and in many cases as an alternative treatment, bismuth and creasote together will be found extremely good. They may be well combined by dropping a minim of the liquid first upon a small quantity of magnesia, rubbing up with eight grains of subnitrate of bismuth, and dividing into four powders; for elder children, into two. They should be freshly prepared for use, and to infants given gently on a moistened finger-tip every three, four, or six hours. In the intervals a little saccharated lime-water with milk should usually be given.

*Chills Viewed in a New Light.*—The wisdom and kindness of Providence in the allotment of disease is not not always appreciated by the suffererers themselves, at least. Mark Twain in his narrative of "Old Times on the Mississippi," in the March *Atlantic*, makes clear, however, the happy and providential aspect of one disease, at least. Describing graphically the life and aspect on the low bottom lands bordering the river, during a big rise and overflow, he says.

"There were crazy rail-fences sticking a foot or two above the water with one or two jeans-clad, chills-rocked, yellow-faced male miserables roasting on the top rail, elbows on knees, jaws in hands, grinding tobacco,and discharging the result at floating chips,through crevices left by last milk-teeth; while the rest of the family, and the few farm animals, were huddled together in an empty wood-flat, riding at her moorings close at hand. In this flat-boat the family would have to cook, and eat, and sleep, for a lesser or greater number of days (or possibly weeks), until the river should fall two or three feet, and let them get back to their log-cabins and their chills again—chills being a merciful provision of an all-wise Providence, to enable them to take exercise without exertion.—*Medical Examiner.*

*Chloral Hydrate.*—Acts first, as an excitant; secondly, as an aæsthetic, especially on the cerebral centers. Its action, usually rapid, depends on its more or less good quality, and on the varying individualities. It can be given either by the mouth or rectum; in the former case, mixed with syrup, in teaspoonful doses, or with water when given in the form of enema. The administration of chloral as a hypnotic is called for in all conditions which require the beneficial effects of recuperating sleep. It is contra-indicated in cardiac debility with valvular lesions; in disorganizations in the mucous membrane of the digestive organs; in advanced diseases of the respiratory organs, etc. The medium dose varies between thirty and seventy-five grains. When given beyond a hundred and twenty grains it acts like a deadly poison. It is an indispensable remedy in the hands of the intelligent physician, but may become a dangerous poison in those of the inexperienced, superficial practitioner.—*Med.-chir. Central.*

*Anilin in Chorea.*—Dr. Fiske (*Pacific Medical Journal*) reports a case cured by this singular remedy. He remarks: "The anilin at first produced a little nausea, which soon subsided. After the fourth day of treatment, it was continued with camphor, made into pills one grain each. In two weeks the case was cured. Dr. Justin reports several cases in which like results followed. Dr. Peter Jones, of Terre Haute, Indiana, has also used it with like success in several cases, notably one which had continued for fifteen years. Dr. Aaron Wilson, of Nachitoches, has also used it, especially among the negro population, with marked success. It is best to commence with half-grain doses of the medicine, gradually increasing till two grains are given for a dose. Of the sulphate, three or four grains, gradually increased to eight or ten grains, may be used. It frequently produces a blueness of the skin, which soon passes away, and need not create any alarm. It has been so prompt and beneficial in its action in the hands of these gentlemen, that its properties should be further investigated, and if found worthy, it should take a higher place in our list of remedies."

*Treatment of Carbuncle.*—Consists in the use of adhesive strips, about three-fourths of an inch in width, so applied as to make concentric pressure upon the part, and leaving a space over the centre, corresponding to the size of the main opening, free, over which was applied a linseed-meal poultice, supported by a roller.

The internal treatment consisted in quiniæ sulph., gr. ii, and ferrum redact., gr. i, given four times daily, with two bottles of ale, and a well-regulated house diet.

*Sulpho-Carbolate of Sodium as a Prophylactic in Scarlatina.*—(*The Medical Press and Circular.*)—Dr. William Scott is desirous of directing attention to the use of the sulpho-carbolate of sodium, both as a curative and prophylactic agent, in the treatment of scarlatina. He has seen its use followed in this disease by results which far exceeded his most sanguine anticipation. In scarlet fever, diphtheria, and measles, the sodium sulpho-carbolate may be given in doses varying, according to age, from five to thirty grains, four times daily, and in a mixture with simple syrup or other such ingredient is in no way disagreeable.

*Anthelmintic Action of Kameela.*—M. Blondeau (*Bull. Gen. de Therap.*) has employed the tincture of kameela with success in cases of tænia. He gave, in one case, f ʒ vj. tincture of kameela in infusion of sage, divided into three doses and taken at intervals of an hour,—at nine, ten, and eleven in the morning. At one o'clock in the afternoon, without having experienced the least colic, the patient voided an enormous tænia, the head of which was unfortunately missing.

This anthelmintic possesses, according to M. Davaine, who also advocates its use, the following advantages: it is not disagreeable to take; it does not cause colic, and it need not be asssociated with a purgative.

Kameela is obtained from the capsules of one of the Euphorbiaceæ, the *Rottlera tinctoria.* It is a red powder used in dyeing silk. Its use as an anthelmintic has been suggested by Hunsby. Anderson, who has also employed the tincture, has never gone beyond the dose of f ʒ iiiss.

*Hydrate of Chloral.*—M. Oré states that the addition of two drops of ten per cent. solution of carbonate of sodium to fifteen grains of chloral dissolved in a drachm of water, will not only neutralize the acidity of the chloral, but will render the solution alkaline. This alkaline solution hinders the coagulation of the blood, instead of occasioning it, and it can therefore be injected into the veins without any risk of producing embolism. M. Oré strongly recommends this method of employing chloral as a means of producing anæsthesia in surgical operations.—*Practitioner;* from *Bull. Gen. de Therap.*

*A New Method of Administering Enemata of Chloral.*—M. Dujardin-Beaumetz has employed the process suggested by Griffith for administering chloral enemata with success. A solution containing a drachm of chloral is beaten up with the yolk of an egg, and this is added to a glass of milk to form the enema. This mixture has the advantage of causing no pain, which is not the case when doses of thirty to sixty grains of chloral are administered in the usual enema or suppository.—*Bull. Gen. de Therap.*—*Medical Times.*

*Eczema.*—Dr. Duhring remarked that eczema varies very greatly, as it occurs, in one part or another of the body, and even in the same part from day to day. The case before the class had been, when seen a few days previously, a case of pure eczema rubrum, but was now, in part at least, eczema squamosum. The treatment would have to be somewhat guarded, as a dispensary patient could not be kept under control in the manner of a hospital patient. The use of the following ointment was directed:

℞. Hydrarg. chlor. mit., . . . ʒi;

Unguent. zinci oxid., . . . ℥ii.—M.

The leg is to be carefully cleansed with hot water and brown soap, morning and evening, and the ointment applied, spread upon soft rags. As the patient suffers from constipation, she will take, at the same time, some simple aperient mixture.

*Hæmatoxylon in Alkalinity of the Urine* (*Medical News and Library.*—Last year M. Cotton published, in the *Lyon Medical*, a communication relative to the antiseptic qualities of this substance. According to him, fermentation cannot take place in an infusion of the wood; and if a few chips are placed in decomposing urine its odor is removed. With the object of ascertaining whether this action could take place in the animal economy, M. Cotton gave to a man aged forty-five, who had suffered twenty-three years from distressing frequency of micturition, his urine being very alkaline from ammonio-magnesian phosphate, and also albuminous, an infusion of half a drachm of logwood night and morning. In ten days the urine had become acid and free from crystals of the triple phosphates. The proportion of albumen was not changed, but there was a marked diminution in the frequency of micturition.

*Asthmatic Mixture.*—The following will relieve the attacks of asthma in a goodly number of cases:

℞—Potass. iodid., . . . . . ʒj.

Vin. ipecac., . . . . . . ʒij.

Spts. ether. co., . . . . . ʒj.

Syr. simplicis., . . . . . . ℥j.

Aquæ, . . . . . . . ℥v.—M.

S. Teaspoonful every two hours during paroxysm.

*Liquor Ferri Perchloridi as a Local Application in Cancerous Ulceration of the Uterus.*—When the course of the cancer is rapid, the application of the strong perchloride of iron is of little use; but when the disease is purely epithelial, or chronic, or rodent in character, it relieves, or sometimes seems to cure bad cases. Its application rarely causes pain. All discharges should be washed away from the cancer, and the breech of the patient raised to prevent any overflow of the solution over the vulva. Cotton-wool saturated with the perchloride is then applied, and any superabundant solution which a slight pressure of the wool causes to flow out, is to be sucked up by a sponge from the bottom of the vagina, The wool is to be retained in its place by a loose plug of tow in the vagina, and the vulva oiled before the patient rises from the couch.—*The Lancet, December* 12, 1874.

*Treatment of Varix by Local Application of Perchloride of Iron.*—Dr. Linou, of Verviers, has, for three years, treated varix with great success by the local application of perchloride of iron. He used a solution of the strength of six grammes to two hundred of water. Compresses of flannel are to be wrung out in this fluid and applied to the parts with a moderately tight bandage. These applications should be renewed for seven or eight days consecutively, after which the bandage should be continued without further wetting with the solution. After a time the solution is again used, and the bandage is then applied and left till the varix has entirely disappeared, which generally occurs in one or two weeks, according to the extent of the swelling.—*L'Independente and Gaz. Med. Ital. Prov. Venete.*

*Whooping-Cough* (*The Cinciannati Lancet and Observer*, October 1875).—Dr. Wild reports in the *Wiener Allg. Mediz. Zeit.* that he has succeeded in curing whooping-cough in eight days by the following treatment: The patient is not allowed to leave the room, and at each fresh paroxysm a compress, folded in several layers and saturated with a teaspoonful of the following solution, is held to the mouth. The patient is thus compelled to inhale:

℞. Ether, 60 parts,
Chloroform, 30 parts,
Turpentine, 1 part.

*Formula for the Combined Administration of Cod-Liver Oil and Phosphorus* (*The Medical Record*, September 18, 1875).—Dr. Edward C. Mourn has employed the following mixture with the happiest results, patients taking it readily who could not bear the plain cod-liver oil at all.

| | | |
|---|---|---|
| ℞. | Yolk of three eggs; | |
| | Olei morrhuæ, . . . . | ℥viij.; |
| | Sherry wine, . . . . . | ℥iv.; |
| | Acidi phosphorici, | |
| | Syrupi simplicis, aa . . . | ℥i.; |
| | Aquæ amygdalæ amaræ, . . | ℥viij.; |
| | Spiriti vini rect., . . . . | ʒi.—M. |

Rub the eggs up in a mortar, adding the oil spoonful by spoonful. Last of all add the phosphoric acid.

*Actual Cautery.*—Dr. Bounafort, of Belgium, has devised the following for application to the neck of the womb: nitrate of potash, one part; wood-charcoal, twenty-eight parts; powdered gum arabic, four parts; water, q. s. Mix thoroughly, and mould into sticks the size of the little finger, and from half to three-quarters of an inch long. When dry, set fire to one end of the little stick, and carry it through a glass or wooden speculum to the exact spot. With proper care the patient is unaware of what has been done. Cold water is then thrown in, a tampon dipped in glycerin is left *in situ* for a month, when, if necessary, the cauterization can be repeated.

*Formula for the Administration of Croton-Chloral Hydrate.*—

| | | |
|---|---|---|
| ℞. | Croton-chloral, . . . . | gr. xxx. |
| | Glycerinæ, . . . . . | ʒiss. |
| | Ext. glycyrrhiz, . . . | ʒi. |
| | Aq., et syr. simpl. aa, . . . | f℥iss.—M. |

Tablespoonful *pro re nata.*

*Pills of the Above.*

| | | |
|---|---|---|
| ℞. | Croton-chloral, | |
| | Pulv. glycyrrhiz., | |
| | Confect. rosarum, aa. . | gr. xv.—M. |

Ft. in pil. No. xx.

*Charcot on the Relief of Hysterical Seizures by Compression of the Ovaries.*—According to Charcot most hysterical seizures are preceded by an aura starting from one or both the ovaries, and he finds that pressure on the organ indicated causes immediate arrest of the seizure. He illustrated this action upon a patient in the Salpetriére affected with hystero-epilepsy. The seizure recurred, however, the moment the pressure was taken off. In order to keep up the pressure for a longer time than would be possible with the unaided hands, he recommends an apparatus like a tourniquet. The pressure is to be made in the situation and direction requisite in compressing the iliac artery, and, in fact, this vessel will be felt pulsating under the finger.—*Gaz. Med. de Paris*, November 5, 1875.—*New York Record*, June 19.—*The Clinic.*

*Crayons of Iodoform.*—Dr. Leblond strongly recommends (*Annales de Gynæcologie*) the crayons of iodoform introduced into therapeutics by M. Gallard, and which are used advantageously in superficial ulcerations of the neck of the uterus, extending into the uterine cavity. These crayons are introduced into that cavity and kept there by a tampon of cotton placed in contact with tne neck of the uterus.

The best formula for these crayons is that of M. Godin, which is as follows: ℞. Very finely powdered iodoform, gr. x; powdered gum arabic, centr. 1; mucilage sufficient to make a pilular mass. Divide into ten equal cylinders four centimetres long, and dry in the air for 24 hours. These cylinders may be easily divided into any desired lengths.—*Le Progrès Med.*

*Grains de Sante.*—These pills have a very large sale in this country, as well as France and Germany. It will rarely be necessary to employ more than two or three to produce a comfortable movement.

| | | |
|---|---|---|
| ℞.—Aloes | . . . . | 100 grains. |
| Jalap, | . . . . | 100 " |
| Rhubarb, | . . . | 25 " |
| Syrup of wormwood | . | sufficient. |

Divide into two-grain granules, which are to be silvered. Dose, one to ten before meals.

*Chloral and Bromide of Potassium in Chest Affections.*—Dr. S. L. Abbott, of Boston, in a communication to the Philadelphia *Medical Times,* says: "With regard to the use of chloral and bromide of potassium together as a sedative in cases of chest affection, I would say that I have repeatedly used this combination, and I dare say it has occurred to many others to employ it. During the present autumn, some of my patients, who are victims of 'autumnal catarrh,' have found great comfort from it. The addition of a little morphia adds to its efficacy, and in one instance, at least, that of a lady who is very susceptible to the disagreeable after-effects which opiates so often produce, the chloral and bromide seem to prevent their occurrence entirely."

*The History of Chloroform.*—A day or two after the meeting in which the announcement of the discovery of the power of chloroform was made, it was arranged to perform a severe surgical operation on a patient while under its influence, and Dr. Simpson was to administer it. At the appointed hour Dr. Simpson could not be found, as he had been suddenly summoned into the country to attend a very important case. The operation, however, was proceeded with, no anæsthetic being used, and the patient died on the operating table. Had chloroform been given in this case the death would undoubtedly have been charged to it, and in all human probability, it would have changed the whole history of anæsthesia by chloroform, if, indeed, it had not permanently caused its abandonment.—*Dunster: Peninsular Journal, August,* 1875.

*Antispasmodic Pills.*—The London *Medical Record* gives the following formula:

| | |
|---|---|
| ℞.—Pulv. assafœtidæ, | |
| Pulv. camphoræ, . . . . | aa. ʒ vj. |
| Ext. belladonnæ, . . . . . | ℈ ij. |
| Pulv. opii, . . . . . . | ℈ j. |
| Syrupi, . . . . . . | q. s. |

M. Ft. in pil. No. clxxx.

One to be taken the first day, two the second, and so on until six are taken daily, or two three times a day. Useful in hysterical and spasmodic nervous affections, in connection with bromide of potassium in doses of ten to fifteen grains.

*Treatment of Lead-colic by Chloroform.*—Chloroform has lately been used in lead-colic, both externally and internally (by inhalation), with advantage. Dr. Barduzzi has employed the following formula with success in one case which had resisted other forms of treatment:

℞.—Chloroformi, . . . . ʒ j.

Syr. acaciæ, . . . . . ℥ ij. ʒ vij.

Aq. destillat., ad., . . fl. ℥ vj. M.

This potion is administered in two parts, with a quarter of an hour interval. It was given three times in thirty-six hours, with the effect of relieving the patient greatly. Inunctions with a chloroform embrocation were at the same time employed on the abdomen.

*Hyperidrosis Relieved by Diachylon Plaster.*—John M. Bigelow, A. M., M. D., (*New York Medical Journal*,) reports having cured a case of excessive sweating of the feet, which had for a long time resisted ordinary treatment, by the application of diachylon plaster. Ordering the patient to take his bed, the plaster was applied in strips twisted around each toe separately, fitting them into the interdigital spaces and completely enveloping the whole foot, so that every portion was in immediate contact with the plaster. These strips were removed every morning, the feet carefully wiped with dry, heated flannel, and new plaster strips applied. After thirteen days of this treatment a cure was effected, and for more than a month there has been no return of the sweating.

*Ipecacuanha by Inhalation* (Murrell, *La France Med*).—This observer advises the use of ipecacuanha by inhalation in the treatment of bronchitis when dyspnœa is present. He claims that its use in this way in winter cough has been attended by striking results. He employs the wine, and usually combines it with not more than one to two parts of water, and uses daily one-half to one drachm of the above-named preparation of the drug.

*Impure Vaccine.*—Dr. L. Letzerich found that vaccine which is infected by diphtheria organism cannot produce pustules, but causes local as well as general diphtheria.—*Virchow's Archives, April*, 1875.

*Mustard Foot-Bath in Urticaria.*—A writer in the *Tribune Medicale* recommends the use of the mustard foot-bath in cases of urticaria. In his own case, after trying innumerable remedies, he was about to abandon all hopes of relief, when, one day, while suffering from a peculiarly aggravated attack of his old enemy, complicated by an excruciating headache, and hoping to relieve the latter, he plunged his feet into a mustard bath. The relief was instantaneous, and it seemed as though the skin-disease had disappeared by a wave of the hand. Five other cases were subsequently treated by the writer with similar reliet. The treament is of course understood to be only palliative, and has no influence in preventing recurrence of the disorder.

*The Facial Discolorations of Pregnancy* (*The St. Louis Clinical Record*, September, 1875).—Dr. E. Dubois treats all his cases with tincture of iodine. The epidermis exfoliates when painted over every evening. If success should not follow the first trials, the treatment must be suspended and the surface dressed with cold cream. Then, when the epidermis is again reproduced, the applications are resumed, and this time the marks always disappear.

*Treatment of Herpes Zoster by Induced Currents.*—Dr. Fanque recommends the use of induced currents in the treatment of zoster, which is now generally admitted to be of nervous origin.

He recommends that the positive pole be placed upon the vertebral column while the negative pole is placed upon the affected portion of skin.

*Oxygen in Congestion of the Brain* (*Boston Journal of Chemistry*). A French physician reports to the Academy a case in which he has succeeded in curing grave congestion of the brain, along with paralysis of the whole right side, by making the patient inhale pure oxygen; from the first inhalation the patient got relief, and motion and sensibility returned by degrees.

A DOCTOR'S wife tried to move him by tears. "Ah," said he, "tears are useless; I have analyzed them. They contain a little phosphate of lime, some chloride of sodium, and water."—*Ex.*.

THE following is an excellent pill for the constipation so common to females of a sedentary habit:

| | | |
|---|---|---|
| ℞.—Aloin, | . . . . | gr. ss. |
| Ferri sulph. exsic., | . . . | gr. iss. |
| Ext. nucis vom., | . . . | gr. ss. |
| Ext. belladonnæ, | . . . | gr. ss. |

Ft. pil. One or two pills daily.—*Medical Times.*

*Hypodermic Injection of Pure Water.*—(*Le Mouvement Medical*, September 4, 1875.)—Dr. Laffitte has employed subcutaneous injections of pure water in a great variety of diseases attended with pain. He has obtained as much success in the relief of this latter element as when he employed injections of morphia, and attributes it to the compression of the nervous terminal filaments by the injected water.

*Harmless Cosmetic Powders.*—The apothecaries of Copenhagen have agreed on the substitution of certain harmless compounds for the numerous poisonous face-powders now commonly used. In avoirdupois weight, the proportions of the ingredients will be about as follows: For white powder, oxide of zinc, 1 oz.; wheat starch, 9 ozs.; oil of rose, 3 drops. For red powder, carmine, 1 oz.; carbonate of magnesia, 4 ozs.

*Sugar-Coated Pills.*—At a late meeting of the Pharmaceutical Society of Philadelphia, Dr. Miller spoke strongly against the purchase of cheap sugar-coated quinia pills. There are in the market forty-five thousand such pills which do not contain a trace of quinia. They were made from muriate of cinchonia, furnished by a New York house as sulphate of quinia to some of the makers of sugar-coated pills.—*New York Evening Post.*

*Valerianate of Zinc in Dysmenorrhœa.*—Dr. Minot, of Boston, (*Boston Medical Journal*) has treated dysmenorrhœa successfully by giving one grain of valerianate of ammonia three times a day, commencing a day or two prior to the advent of the monthly period. When there was tenderness in the region of the ovary, tincture of iodine was applied externally.

Muriatic acid, properly diluted, forms an excellent bleaching substance, and the pharmacist may avail himself of its use with advantage for many purposes. There is no better or more convenient article for removiug stains from the hands and from mortars than this acid.

The store towels, which so often become stained, and present a very untidy and unsightly appearance even after they have been washed, may be greatly improved, if not entirely restored to their original color, by immersing them for a few minutes, after they have been thoroughly washed, in a mixture of one part of muriatic acid to nineteen parts of boiling water, and then thoroughly rinsing them in clean water.

*A Simple Method of Removing the Teeth of Children.*—The operation consists in simply slipping a rubber ring over the tooth and forcing it under the edge of the gum. The patient is dismissed and told not to remove the appendage, which in a few days loosens the tooth and causes it to fall out. Grown children, who shrink from the shock and pain of the dental nippers, may also have their teeth removed by means of the rubber, which is a mild form of treatment.—*Druggists' Circular.*

*Elegant Ferruginous Preparation.*—The following offers simply the most elegant and efficient ferruginous preparation we know of:

Take of tincture of the chloride of iron three fluid drachms, dilute phosphoric acid half a fluid ounce, syrup of lemons three fluid ounces. M. A whitish preparation, pleasant to the taste; to be exhibited in a dose of a dessertspoonful to a tablespoonful.—*Medical Times.*

*Cure for Warts.*—Lisfranc immerses the parts on which the warts are developed in strong solution of black soap. This causes a slight cauterization of the surface of the wart. The loosened tissue is to be removed and the application repeated every day till the cure is complete.

*New Diaphoretic.*—This is the Aabec bark, and is used in the form of fl. ext. or infusion. Of the fluid extract a teaspoonful is given every hour. It is under trial.

